# HIV Alters Gap Junction-Mediated Intercellular Communication in Human Brain Pericytes

**DOI:** 10.3389/fnmol.2017.00410

**Published:** 2017-12-12

**Authors:** Hyung Joon Cho, Alyce Mei-Shiuan Kuo, Luc Bertrand, Michal Toborek

**Affiliations:** Department of Biochemistry and Molecular Biology, University of Miami Miller School of Medicine, Miami, FL, United States

**Keywords:** pericytes, brain microvessels, HIV infection, gap junctions, connexin 43

## Abstract

Despite successful control of viremia by combined antiretroviral therapy, brain infection and its resulting neurocognitive impairment remain a prevalent comorbidity in HIV infected individuals. HIV invades the brain early in the course of infection via penetration through the blood-brain barrier (BBB). While the impact of HIV on BBB astrocytes and endothelial cells is relatively well studied, the role of pericytes in BBB regulation during HIV infection remains unclear; however, it is known that a selective population of pericytes is prone to infection. In the present study, we hypothesize that injury signals are propagated from infected pericytes to neighboring cells via gap junction (GJ)-mediated intercellular communication. Among a variety of studied GJ proteins, HIV infection of human brain pericytes specifically increased expression of connexin 43 as determined by immunoblotting and immunostaining. This effect was confirmed in the brains of mice infected with EcoHIV, a mouse-specific HIV strain. In addition, HIV infection enhanced functional GJ-mediated intercellular communication in pericytes. The importance of this process was confirmed in experiments in which inhibition of GJs by carbenoxolone attenuated HIV infection. In addition to GJs, an extracellular ATP release assay revealed that HIV may also play a role in opening of connexin (Cx)-containing hemichannels (HCs). Overall, these findings indicate an important role of GJs in the propagation of HIV infection in human brain pericytes that may contribute to BBB dysfunction in brain infection and the pathogenesis of NeuroAIDS.

## Introduction

Human immunodeficiency virus (HIV) enters the central nervous system (CNS) shortly after infection by penetrating the blood-brain barrier (BBB) and evokes neurocognitive and motor function deficits, collectively known as HIV-associated neurocognitive disorders (HAND; Cysique et al., 2004; Heaton et al., 2010; Jernigan et al., 2011; Letendre, 2011; Saylor et al., 2016). Despite efficient repression of viral replication by effective antiretroviral treatment, neurocognitive impairment still develops in approximately 40%–60% HIV-positive patients (McArthur, 2004; Heaton et al., 2011; Letendre, 2011). In fact, recent findings indicate that the prevalence of HAND is increasing, partly due to the longer life span of HIV-infected individuals and the increasing number of patients resistant to antiretroviral therapy (Nath and Sacktor, 2006; Ances and Ellis, 2007; Saylor et al., 2016). Therefore, it is of critical significance to unravel the mechanisms involved in the pathogenesis of brain infection by HIV. Previous studies reported the effects of HIV and HIV-1-specific proteins on various cell types of the BBB, including endothelial cells and astrocytes (Cosenza et al., 2002; Toborek et al., 2005; Eugenin et al., 2011; Strazza et al., 2011; Berman et al., 2016; Sami Saribas et al., 2017). However, the role of pericytes in brain infection by HIV has been largely overlooked and not yet extensively examined.

Structurally, BBB pericytes reside in the sub-endothelial space of microvessels and cover about 30% of the abluminal surface of endothelial cells, although the precise identity of pericytes and their cell lineage are still controversial (Mathiisen et al., 2010; Dalkara et al., 2011; Hill et al., 2014). BBB pericytes have recently attracted attention given the vital and active nature of their newly identified role in the maturation and maintenance of the BBB by controlling permeability, angiogenesis, cerebral blood flow and neuroinflammation (Armulik et al., 2010; Bell et al., 2010; Winkler et al., 2011; Hill et al., 2014; Sweeney et al., 2016). In addition, previous studies demonstrated their important role in diverse neurological diseases (i.e., Alzheimer’s disease, amyotrophic lateral sclerosis, and brain cancers; Heldin, 2013; Sagare et al., 2013; Winkler et al., 2013) and their ability to be infected by HIV (Nakagawa et al., 2012; Castro et al., 2016).

Previously, we demonstrated that BBB pericytes express the chemokine receptors CXCR4 and CCR5, two major co-receptors for HIV-1 entry, and that their infection may contribute to the progression of HIV-induced CNS damage by causing BBB disruption and increasing endothelial permeability (Nakagawa et al., 2012). It has been suggested that regulatory processes at the BBB are, at least in part, dependent on extracellular interactions via soluble factors and direct intercellular communication through gap junctions (GJs; Bell et al., 2010; Eugenin et al., 2011; Winkler et al., 2011). We hypothesize that similar processes are involved in pericyte-mediated BBB regulation; therefore, the present study focuses on GJ-mediated intercellular communication in HIV-1 infection of BBB pericytes.

GJ channels directly link neighboring cells by providing the pathway for intercellular communication via exchange of ions and signaling molecules. GJs are comprised of connexins (Cxs; Leithe et al., 2012), which also constitute the protein component of hemichannels (HCs) that are responsible for communication between the cell and its environment (Vega et al., 2013). Various Cxs are present in all cell types of the neurovascular unit and play important roles in cell-cell communication throughout the CNS (Laird, 2010). While our understanding of Cx functions in various pathological conditions is far from complete, emerging evidence suggests that functional alterations of Cxs may be related to pathogenesis of several CNS diseases, including brain infection by HIV (Matsuuchi and Naus, 2013; Eugenin, 2014).

An unexplained phenomenon in brain infection by HIV is that the virus infects a relatively limited population of CNS cells; however, neuropathology and neurocognitive dysfunction are prominent (Gannon et al., 2011; Kovalevich and Langford, 2012; Saylor et al., 2016). Indeed, our *in vitro* studies indicate that HIV infects only ~5% of pericyte populations (Nakagawa et al., 2012). In the present study, we hypothesize that GJ can participate in the propagation of injury signals from a small number of infected pericytes to neighboring cells. The study is novel as there are no reports on the impact of HIV infection on pericyte cell-to-cell communication via junctional complexes.

## Materials and Methods

### Cell Cultures

Primary human brain vascular pericytes from four different lot numbers (ScienCell, Carlsbad, CA, USA) were cultured in complemented growth medium (ScienCell) with fetal bovine serum (FBS; 2%) and growth supplement containing 100 units/mL penicillin and 100 μg/mL streptomycin. Human embryonic kidney (HEK) 293T/17 (ATCC, Manassas, VA, USA) cells were grown in DMEM (Thermo Fisher Scientific–Gibco™, Carlsbad, CA, USA) supplemented with 10% FBS (Thermo Fisher Scientific–Gibco™) containing 100 units/mL penicillin and 100 μg/mL streptomycin. All cells were cultured at 37°C with 5% CO_2_ and 95% air under a humidified atmosphere.

### HIV Production, Quantitation and Infection

HIV-1 (strains NL4-3, YU-2, or 49.5) were obtained from the NIH AIDS Reagent Program, and EcoHIV (NL4-3 or NDK) were kindly provided by Dr. David Volsky (Mount Sinai Hospital, New York, NY, USA). Viral stocks were produced by transfection of HEK293T/17 cells with 50 μg of proviral DNA plasmids using Lipofectamine 3000 (Thermo Fisher Scientific-Invitrogen™, Carlsbad, CA, USA) according to manufacturer’s instructions. Supernatants containing virus were collected 48 h after transfection, filtered (0.45 μm-pore size filter), quantified by HIV type 1 p24 ELISA kit (ZeptoMetrix, Buffalo, NY, USA), and stored at −80°C until use. Confluent cultures of human pericytes were infected by incubation with viral stocks (60 ng of p24/ml/1 × 10^6^ cells) for 24 h, followed by extensive washing with PBS to remove the unbound virus before addition of fresh medium. For specific control experiments, HIV was inactivated by exposure to UV light 100 μW/cm^2^ for 30 min.

### EcoHIV Infection and Isolation of Brain Microvessels

All animal procedures were approved by the University of Miami Institutional Animal Care and Use Committee in accordance with the NIH guidelines. Male C57BL/6 mice (12-week-old, Jackson Laboratories, Bar Harbor, ME, USA) were acclimatized to the animal facility for 1 week with free access to food and water. Animals were then infused with EcoHIV/NDK (1 μg of p24) through the internal carotid artery using a method previously described (Bertrand et al., 2016; Leda et al., 2017), while control animals received saline. Mice were sacrificed at day 7 post-infection. Brains were quickly removed after transcardial perfusion, immediately immersed in ice-cold PBS, and homogenized in ice-cold isolation buffer (102 mM NaCl, 4.7 mM KCl, 2.5 mM CaCl_2_, 1.2 mM KH_2_PO_4_, 1.2 mM MgSO_4_, 15 mM HEPES, 25 mM NaHCO_3_, 10 mM glucose, and 1 mM sodium pyruvate with Halt™ proteinase inhibitor cocktail; Thermo Fisher Scientific-Pierce™, Rockford, IL, USA). Then, samples were filtered through a 300 μm mesh filter (Spectrum Laboratories, Rancho Dominguez, CA, USA), and transferred to centrifuge tubes containing 26% dextran (M.W. 75,000) in isolation buffer solution. The samples were centrifuged at 5800× *g*, 20 min, at 4°C, the supernatants were discarded, pellets were resuspended, filtered through a 120 μm mesh filter (Millipore Sigma, Billerica, MA, USA), and submitted for another round of centrifugation at 1500× *g*, for 10 min, at 4°C. The supernatant was removed, and the pellet, containing brain microvessels, was resuspended in 200 μl of isolation buffer. This microvessel-enriched fraction was smeared onto a glass slide (approximately 7 μl per slide), heat-fixed at 98°C for 10 min, and used for confocal microscopy analysis.

### Droplet Digital Polymerase Chain Reaction for Quantifying HIV DNA Integration

Cellular DNA was extracted using the QIAamp^®^ DNA mini kit (Qiagen, Valencia, CA, USA) according to the manufacturer’s protocol. The concentration of DNA was estimated using a NanoDrop 2000 spectrophotometer (Thermo Fisher Scientific, Wilmington, DE, USA) and the A_260_/A_280_ absorptivity ratio. Primers and fluorogenic probes were designed to quantitate the total HIV-1 clade B genome, Rtotal primer: 5′-CTG CAT CCG GAG TAC TTC AAG AAC TG-3′; C1r primer: 5′-TCC CAG GCT CAG ATC TGG TCT A-3′, 2n4n probe: 5′-AGT GGC GAG CCC TCA GAT GCT GC-3′ (Sharkey et al., 2000). All targets were measured by droplet digital polymerase chain reaction (ddPCR) using a Bio-Rad QX100 ddPCR instrument (Bio-Rad, Hercules, CA, USA), which provides a dynamic range of detection from 1 to 10^5^ copies. All of the target amplicons were cloned into plasmids and used as positive controls to validate the assay. The single-copy human CCR5 gene was quantified to measure the number of cell equivalents in DNA samples for standardization (Sharkey et al., 2000).

### Occludin Depletion

Occludin was silenced by transfection (Lipofectamine RNAiMAX; Thermo Fischer Scientific-Invitrogen™) with 25 nM anti-occludin 27-mer human siRNA (OriGene, Rockville, MD, USA) in RNA resuspension buffer (100 mM KAc and 30 mM HEPES; pH 7.5). Trilencer-27 universal scrambled (SCR) negative control siRNA (OriGene, Rockville, MD, USA) was used as a control.

### Immunostaining and Immunoblotting

Cells or brain microvessels were fixed with 4% (v/v) paraformaldehyde for 15 min at room temperature, rinsed with PBS, and permeabilized in 0.1% (v/v) Triton X-100 for 10 min. After being washed with PBS, non-specific binding sites were blocked with 3% (w/v) bovine serum albumin (BSA) in PBS for 1 h at room temperature. Then, samples were incubated overnight at 4°C with the primary antibody, rabbit (1:100, Cell Signaling Technology, Danvers, MA, USA) or mouse (1:100, Abcam, Cambridge, MA, USA) anti-Cx43, rabbit anti-PDGFRβ (1:100, Cell Signaling Technology), or mouse anti-HIV1 p24 (1:100, Abcam), diluted in 1% (w/v) BSA in PBS. Samples were washed with PBS and incubated with secondary antibodies, goat anti-mouse or anti-rabbit IgG conjugated with Alexa Fluor 488 or 594 (Thermo Fischer Scientific-Invitrogen™; 1:400 dilution in PBS) in the dark for 2 h. Hoechst (Thermo Fischer Scientific-Invitrogen™) was employed to stain cell nuclei. In addition, Vybrant^®^ CM-DiI (5 μM for 2 h at 37°C; Thermo Fisher Scientific-Molecular Probes™, Eugene, OR, USA) was used to visualize the cellular membrane for long-term preservation of dyes in cells throughout fixation and permeabilization procedures. Images were acquired with either Olympus FLUOVIEW 1200 Laser Scanning Confocal Microscope (Olympus, Center Valley, PA, USA) or Nikon Eclipse Ti-U Inverted Fluorescence Microscope (Nikon Instruments Inc. Melville, NY, USA), and analyzed using ImageJ software (NIH, Bethesda, MD, USA). Mean fluorescence intensity of the target protein was calculated by integrated fluorescence density per unit area in each image.

For immunoblot analysis, the cells were lysed in RIPA buffer (Thermo Fisher Scientific-Pierce™) containing protease/phosphatase inhibitors (Thermo Fisher Scientific-Pierce™), and protein concentration was assessed with the BCA protein assay kit (Thermo Fisher Scientific-Pierce™). Samples diluted in Laemmli SDS sample buffer (Boston Bioproducts, Ashland, MA, USA) were loaded onto 10% or 4%–20% Tris glycine extended (TGX) precast gels (Bio-Rad) and transferred to nitrocellulose membranes using a Trans-Blot Turbo System (Bio-Rad). Afterward, the membranes were blocked with 4% BSA in TBS. Primary antibodies were incubated overnight at 4°C in blocking buffer supplemented with 0.1% Tween-20 and applied at 1:1000 (mouse anti-Cx43, Abcam; rabbit anti-phospho-Cx43 [S368], Cell Signaling Technology). Membranes were imaged in a LI-COR CLX imaging system with the secondary antibodies diluted at 1:20,000 (anti-mouse 800CW and anti-rabbit 680RD). Target protein levels were normalized to GAPDH using anti-GAPDH conjugated with Dylight 680 (1:20,000; Novus Biologicals, Littleton, CO, USA), and phosphorylated forms of target protein levels were normalized to their total levels. Signal quantification was performed using Image Studio 4.0 software (LI-COR).

### Dye-Coupling Assay and Adenosine-5′-Triphosphate (ATP) Release Assay

Functional assay of the GJ-mediated intercellular communication was performed with a dye-loading technique by means of immunofluorescence or flow cytometry. Briefly, confluent human primary brain pericytes (donor cells) were loaded with calcein acetoxymethyl ester (Calcein-AM; 1 μM for 30 min at 37°C; Thermo Fisher Scientific-Molecular Probes™), while similar cultures of recipient pericytes were labeled with Vybrant^®^ DiI (for immunostaining studies) or DiD (for FACS; both at 5 μM for 30 min at 37°C; Thermo Fisher Scientific-Molecular Probes™). After dye loading, donor cells were trypsinized and added to a monolayer of recipient cells at a ratio of 1:5. Carbenoxolone (CBX; 100 μM; Tocris Bioscience, Minneapolis, MN, USA), a non-specific connexin blocker, was added to the co-culture to ascertain that the dye transfer from the donor to recipient pericytes was dependent on GJ/Cx channels. Donor cells were allowed to attach to the monolayer of recipient cells for 3 h at 37°C, trypsinized, resuspended in PBS containing 5% FBS, and analyzed for dye transfer by immunofluorescence or the BD FACS Aria-IIu cell sorter (BD Biosciences, San Jose, CA, USA). For flow cytometry analysis, DiD (Ex 644 nm; Em 665 nm), a lipophilic tracer similar to DiI (Ex 549 nm; Em 565 nm), was employed to better separate cells labeled with calcein (Ex 495; Em 520 nm).

To assess hemichannel activity, an ATP release assay was performed using an ATP bioluminescent assay kit (Promega, Madison, WI, USA) and calculated based on a luminescence standard curve.

### Statistical Analysis

All statistical analyses were performed with GraphPad Prism 6 (GraphPad Software, La Jolla, CA, USA). One-way ANOVA with Holm-Sidak’s multiple comparisons or two-tailed student’s *t*-test was used to compare mean responses with control groups. Statistical probability of *p* < 0.05 was considered significant.

## Results

### HIV-1 Replication in Human Primary BBB Pericytes

**Figure 1:**
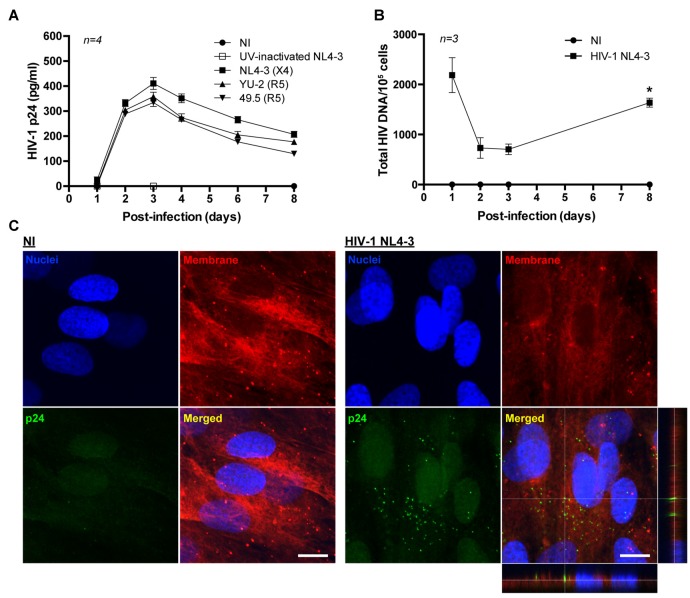
HIV-1 replication in human primary blood-brain barrier (BBB) pericytes. **(A)** Quantification of p24 release into culture media at the indicated days post-infection with different X4 and R5 tropic HIV strains. Data represents mean ± SEM for a representative experiment (*n* = 4) from three independent experiments (total *n* = 12 per group). No p24 levels were detected in non-infected (NI) group and UV-inactivated HIV NL4-3. **(B)** HIV DNA integration into the genome of human primary brain pericytes as quantified by droplet digital PCR (ddPCR). Cells were infected with HIV-1 NL4-3. *p < 0.05 vs. Day 3 post-infection. **(C)** Representative images of p24 immunoreactivity at day 2 post-infection in NI and HIV-1 NL4-3 infected groups (orthogonal view in the merged image of HIV-1 NL4-3 group). No p24 levels were detected in NI group. Nuclei (blue, Hoechst staining), p24 (green, HIV marker) and membranes (red, DiI staining). Scale bar: 10 μm.

Brain pericytes were infected with either X4 or R5 tropic HIV-1 strains (X4 tropic: NL4-3; R5 tropic: YU-2 and 49.5) by a 24 h incubation with the virus. The maximum viral replication as detected by medium p24 levels was observed at day 3 post-infection, followed by decline to the baseline levels at day 8 post-infection (Figure 1A). Infection with the control, UV-inactivated HIV-1 (NL4-3) demonstrated no p24 levels at day 3 post-infection. Moreover, HIV-infected brain pericytes were visualized by the presence of p24-positive cells via confocal microscopy analyses. Figure 1C illustrates the representative images at day 2 post-infection.

Total HIV-1 DNA in infected pericytes was quantified by ddPCR. Integration of the HIV genome with the pericyte genome exhibited an inverse correlation with the levels of HIV-1 p24 collected from the culture media. Specifically, the lowest levels of integrated HIV DNA were observed at days 2–3 post-infection, when medium p24 concentrations were the highest. Subsequently, HIV DNA levels increased, correlating with a decline in p24 levels in the culture media (Figure 1B). Such patterns of change suggest that human brain pericytes might form HIV reservoirs.

### HIV Infection Selectively Up-Regulates Cx43 in Brain Pericytes

**Figure 2:**
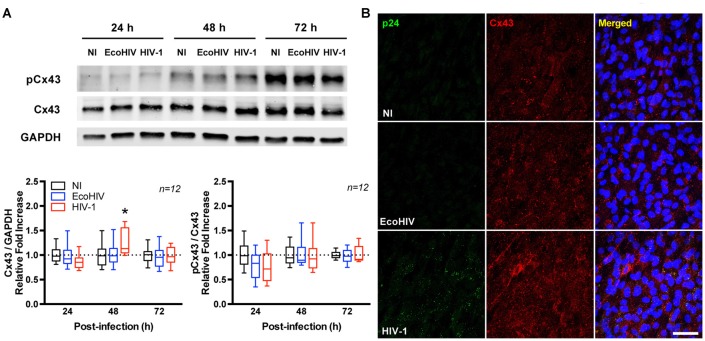
HIV infection up-regulates connexin 43 (Cx43) expression. Pericytes were infected with HIV-1 NL4-3. **(A)** Total and phosphorylated Cx43 protein levels at 24, 48 and 72 h post-HIV infection. Upper panel shows representative Western blots and lower panel reflects quantitative results of these analyses as compared to the NI group. Total Cx43 levels were normalized to a reference target (GAPDH) while pCx43 levels were normalized to total Cx43. EcoHIV does not infect human cells; therefore, it was used as negative control. Box-and-whisker plots display the median within the interquartile range box, with whiskers extending to the minimum and maximum data values (*n* = 12 from three independent experiments). *p < 0.05 vs. NI. **(B)** Representative images of Cx43 distribution in HIV-infected BBB pericytes or controls (NI or EcoHIV). Infected cells (i.e., cells expressing HIV-1 p24) are characterized by more intense Cx43 immunoreactivity compared to controls. Scale bar: 50 μm. pCx43: phosphorylated Cx43.

To determine the impact of HIV on connexin levels, Cx40, Cx43 and Cx45 protein expression was assessed in infected BBB pericytes. Among studied Cxs, HIV selectively up-regulated total Cx43 protein levels at day 2 post-infection as determined by immunoblotting (1.3-fold increase over control levels; Figure 2A) and immunofluorescence staining (Figure 2B), while no significant alterations were found in Cx40 and Cx45 expression (data not shown). The expression of phosphorylated Cx43 (pCx43) was proportional to the up-regulated total Cx43. Thus, when normalizing the levels of pCx43 to total Cx43, no statistically significant changes were observed between the groups.

Control experiments were performed using EcoHIV (Potash et al., 2005; Saini et al., 2007), a modified strain of HIV expressing the attachment protein gp80 (the envelope of ecotropic murine leukemia virus), which infects mouse cells but not human cells. Nevertheless, EcoHIV stock was prepared and cells were treated the same way as with HIV-1. Therefore, treatment of human cells with EcoHIV allows to account for any potential non-specific effects. As illustrated and quantified in Figure 2A (Supplementary Figure S1), exposure of human BBB pericytes to EcoHIV did not alter Cx43 protein expression, indicating specificity of responses.

**Figure 3:**
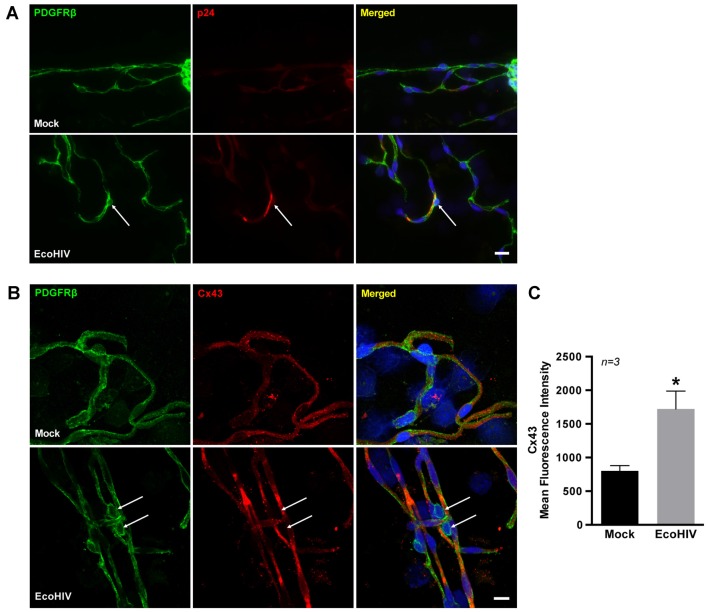
Infection of BBB pericytes and Cx43 expression in EcoHIV-infected brains. **(A)** Representative images of pericytes and p24 distribution in brain microvessels isolated from mock or EcoHIV-infected mice. PDGFRβ (green; arrows) was used as a marker for pericytes, and p24 (red; arrows) immunoreactivity as a marker of active HIV infection. Scale bar: 25 μm. **(B)** Immunofluorescence images of pericytes stained for PDGFRβ (green) and Cx43 (red) distribution in brain microvessels isolated from mock or EcoHIV-infected mice. Cx43 expression was visibly up-regulated (arrows) in microvessels isolated from EcoHIV-infected brains. Scale bar: 10 μm. **(C)** Quantification of mean fluorescence intensity signal for Cx43 analyzed in microvessels. Data were obtained from 3 mice per group, 6–10 microvessels per mice. Mean fluorescence intensity was calculated by integrated fluorescence density/area. Data represents mean ± SEM (*n* = 3). *p < 0.05 vs. Mock.

We next evaluated Cx43 protein expression in BBB pericytes of mice infected with EcoHIV. At day 7 post-infection, mice were sacrificed and brain microvessels were isolated and stained for platelet-derived growth factor receptor β (PDGFRβ; a pericyte marker) and p24 (an indicator of active infection). While a faint background p24 immunoreactivity in mock-infected mice was detected, the merged images clearly indicated p24 positive staining in BBB pericytes in EcoHIV-infected mice (Figure 3A, arrows). To the best of our knowledge, this is the first evidence that brain pericytes can be infected *in vivo*. In addition, Cx43 expression levels were significantly increased in brain microvessels isolated from EcoHIV-infected mice compared to those from mock-infected mice (Figure 3B). Quantitative analysis of signal intensity by mean fluorescence intensity demonstrated a significant increase of Cx43 in EcoHIV-infected mice (Figure 3C).

### HIV Infection Increases Functional GJ-mediated Communication in BBB Pericytes

**Figure 4:**
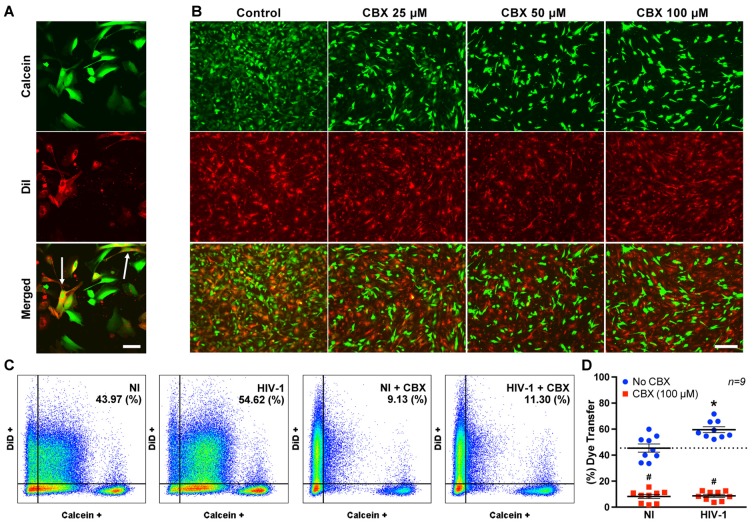
HIV infection enhances gap junction (GJ) channel function. **(A)** Representative images of the dye-coupling technique employed as a functional assay to assess GJ-mediated channel function. Donor pericytes were labeled with calcein (green), recipient pericytes with DiI (red), and then both cell populations were co-cultured for 3 h. Presence of double-labeled cells (i.e., arrows) indicates dye transfer from donor to recipient cells. Scale bar: 25 μm. **(B)** Representative images of dye-coupling technique as in **(A)** with and without inhibition of connexins by carbenoxlone (CBX). CBX visually decreased calcein transfer from the donor to recipient pericytes as demonstrated by a diminished number of double-positive cells. Scale bar: 200 μm. **(C)** Pericytes were infected with HIV-1 as in Figure 2, and/or exposed to CBX (100 μM), and dye-coupling was assessed by flow cytometry. The analyses were performed at day 2 post-infection. Donor pericytes were labeled with calcein, and recipient pericytes with DiD. The numbers in the upper right corners indicate the percentage of double-positive cells. **(D)** Quantitative analysis of the flow cytometry results. Scatter dot plots show individual values with mean ± SEM (*n* = 9 from three independent experiments). *p < 0.05 vs. NI. ^#^*p* < 0.05 vs. no CBX in NI or infected cells.

A dye-coupling assay was employed to evaluate the impact of HIV infection on GJ-mediated intercellular communication. Figures 4A,B illustrate the principle of this assay. Donor pericytes were stained with calcein-AM (a GJ-permeable dye, producing green fluorescence after being intracellularly hydrolyzed by non-specific esterases) and co-cultured for 3 h with DiI (a lipophilic plasma membrane dye) stained recipient pericytes (Figure 4A). Double-labeled (calcein plus DiI; arrows in Figure 4A) cells indicate transfer of calcein from donor to recipient cells. To prove that the observed effects are GJ-related, the experiments were performed in the presence of CBX, a potent pharmacological inhibitor of Cxs. CBX at the concentrations of 25, 50 and 100 μM markedly protected against calcein transfer from donor to recipient pericytes (Figure 4B).

Flow cytometry was then used to quantify the impact of HIV infection on functional GJ-mediated functions (Figures 4C,D). In these experiments, DiD, instead of DiI, was employed for staining of recipient cells. As indicated, dye transfer in HIV-infected cultures was significantly higher (by ~24%) than in non-infected (NI) cultures. Co-treatment with CBX (100 μM for 3 h) markedly reduced dye transfer in both NI and HIV-infected brain pericytes (both ~79% reduction; Figures 4C,D). These results suggest that HIV infection increases GJ-mediated intercellular communication via Cxs.

### Regulatory Role of Occludin in Cx43 Expression and GJ-mediated Communication in HIV-infected Brain Pericytes

**Figure 5:**
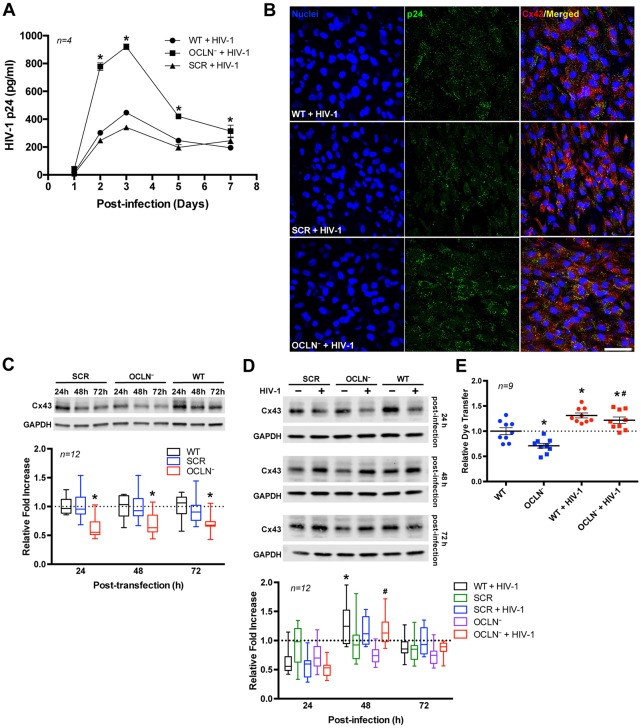
Occludin depletion enhances HIV infection but decreases GJ channel function. BBB pericytes were infected with HIV-1 as in Figure 2 after occludin silencing (OCLN^−^) by transfection with specific siRNA. **(A)** Impact of occludin depletion on the kinetics of HIV-1 infection as measured by p24 levels in the media. Data represents mean ± SEM for a representative experiment (*n* = 4) from three independent experiments (total *n* = 12 per group). *p < 0.05 vs. HIV-1. **(B)** Representative images of p24 immunoreactivity in HIV-infected and/or occludin-depleted BBB pericytes. **(C)** Impact of occludin depletion on Cx43 protein expression in BBB pericytes as measured by immunoblotting *p < 0.05 vs. wild-type (WT) or scrambled negative control (SCR) treated. **(D,E)** HIV infection overcomes **(D)** the impact of occludin depletion on Cx43 protein expression and **(E)** a decrease of GJ channel function as measured by dye coupling (day 2 post-infection). *p < 0.05 vs. WT; ^#^*p* < 0.05 vs. OCLN^−^. Box-and-whisker plots display the median within the interquartile range box, with whiskers extending to the minimum and maximum data values and scatter dot plots show individual values with mean ± SEM (**C**,**D**: *n* = 12; **E**: *n* = 9 from three independent experiments).

Occludin is one of the major tight junction proteins, involved in the maintenance and metabolic regulation of the BBB. The importance of occludin in HIV infection was recently reported by us (Castro et al., 2016). To illustrate this effect, occludin was depleted in BBB pericytes, followed by HIV infection. Then, HIV-1 p24 levels were periodically measured in cell culture media as the indicator of active infection. Occludin depletion significantly elevated the levels of p24 in infected cultures by over 2-folds at day 2 and 3 and over 1.5-folds at days 5 and 7 post-infection (Figures 5A,B).

We next examined the impact of occludin depletion on Cx43 protein expression. As shown in Figure 5C (Supplementary Figure S2), occludin silencing was associated with a decrease in Cx43 protein expression. However, a concurrent HIV infection protected against a Cx43 decrease in occludin-depleted cells as examined 48 h and 72 h after infecting the cells (Figure 5D; Supplementary Figure S3). Similar to results of Cx43 expression, the dye-coupling assay conducted after occludin depletion demonstrated an approximately 29% decrease in GJ-mediated intercellular transfer (Figure 5E). Nevertheless, in occludin-depleted and HIV-infected pericytes this decrease was not observed; in contrast, dye transfer was slightly but significantly increased (~22%) compared to the levels in wild-type (WT) pericytes.

### Functional Inhibition of GJs Protects against HIV Infection of BBB Pericytes

**Figure 6:**
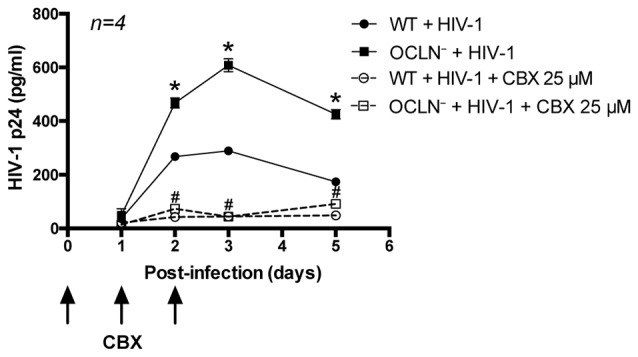
Impact of GJ-mediated intercellular communication on HIV infectivity. BBB pericytes were infected with HIV-1 after occludin silencing (OCLN^−^) by transfection with specific siRNA as in Figure 5. In addition, cells were exposed to CBX (25 μM, GJ blocker), added at the time of infection and days 1 and 2 post-infection. Levels of p24 in the culture media were assessed as in Figure 1. Data represents mean ± SEM for a representative experiment (*n* = 4) from three independent experiments (total *n* = 12 per group). *p < 0.05 vs. WT + HIV-1, and ^#^*p* < 0.05 vs. OCLN^−^ + HIV-1.

To examine whether HIV-infected BBB pericytes use GJs to spread HIV infection to adjacent cells, cultures were exposed to CBX (25 μM), added on the day of infection and days 1 and 2 post-infection. Such dosing allowed us to avoid CBX toxicity, which otherwise may develop in cultures exposed to higher levels of CBX for an extensive time. Blocking GJ-mediated communication dramatically reduced HIV replication in both WT and occludin-depleted pericytes. The maximal levels of p24 (mean ± SEM) in the cell culture media of pericytes infected with HIV in the presence of CBX was 44.2 ± 6.4 (pg/ml) in WT and 43.4 ± 3.9 (pg/ml) in occludin-depleted pericytes. Such levels constituted an 84.7% and 92.9% decrease, respectively, as compared to infected cells with fully functional GJs (Figure 6). These results indicate that GJs are involved in potentiation of HIV infection of BBB pericytes.

### Impact of HIV Infection on Hemichannel Activity

In addition to GJ, Cx-containing HCs play an important role in cell-cell communication. Therefore, the activity of HCs was also evaluated in the current study. These experiments took advantage of the fact that intracellular ATP is released to cell culture media via a process mediated by HCs. Thus, extracellular levels of ATP can serve as markers of hemichannel activity.

**Figure 7:**
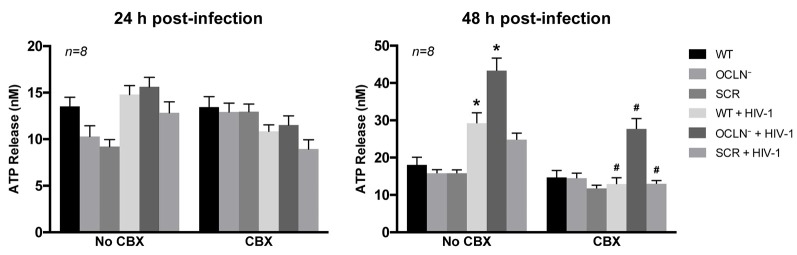
HIV infection opens Cx-containing hemichannels (HCs) in human brain pericytes. BBB pericytes were occludin depleted, infected, and/or exposed to CBX as in Figure 6. ATP levels in cell culture media were assessed by ELISA. Analyses were performed at 24 h or 48 h post-infection. A significant increase in extracellular ATP levels was observed at 48 h post-infection in WT or occludin-depleted pericytes (1.6- or 2.4-fold, respectively) compared with the basal level in NI cells. Extracellular ATP was drastically reduced by additional treatment with CBX (25 μM). Data represents mean ± SEM (*n* = 8 from three independent experiments). *p < 0.05 vs. WT; ^#^*p* < 0.05 vs. no CBX for each treatment.

HIV infection resulted in a significant elevation of ATP release at 48 h post-infection compared to control levels in NI pericytes. This effect was further enhanced by depletion of occludin. Release of intracellular ATP was drastically reduced by addition of CBX (25 μM; Figure 7) to validate the involvement of Cx-containing HCs. These results suggest that HIV infection affects not only GJ-mediated, but also hemichannel-mediated intercellular communication in BBB pericytes.

## Discussion

Recent studies have shed new light on the pivotal role of human brain pericytes in CNS dysfunction, including ischemic brain injury as well as neurocognitive and neurological diseases, such as Alzheimer’s disease, amyotrophic lateral sclerosis brain cancer and HIV-induced neurocognitive deficits (Song et al., 2005; Winkler et al., 2011; Halliday et al., 2016; Sweeney et al., 2016). Pericytes receive signals from neighboring cells and produce functional responses for regulating key neurovascular functions, including BBB formation and maintenance (Armulik et al., 2010; Daneman et al., 2010; Vandenhaute et al., 2012), vascular stability and angiogenesis (Armulik et al., 2011; Winkler et al., 2011; Stapor et al., 2014), clearance of toxic cellular byproducts (Dore-Duffy, 2008; Winkler et al., 2011; Sagare et al., 2013; Pieper et al., 2014), cerebral blood flow (Peppiatt et al., 2006; Fernández-Klett et al., 2010; Hall et al., 2014) and neuroinflammation (Kovac et al., 2011; Jansson et al., 2014; Persidsky et al., 2016). Previously, we showed that human BBB pericytes express the major HIV receptor CD4 and co-receptors CXCR4 and CCR5, which results in their infection by HIV and compromises BBB integrity via functional alterations of endothelial cell tight junction proteins (Nakagawa et al., 2012). Furthermore, occludin depletion induced a robust increase in HIV transcription via regulation of the class III histone deacetylase sirtuin (SIRT)-1/NFκB-p65 acetylation (Castro et al., 2016). However, the involvement of GJ-intercellular communication in HIV infection of human BBB pericytes has been poorly investigated and understood. Intercellular communication is a potentially important problem in HIV CNS pathogenesis, as only a limited amount of pericytes are infected by HIV; yet, the disruption of BBB integrity appears wide-spread (Nakagawa et al., 2012; Castro et al., 2016). Our overall hypothesis is that injury signals from infected pericytes propagate into neighboring NI cells via GJs, enhancing cell toxicity and HIV-induced BBB disruption.

*In vitro* experiments with human BBB pericytes included control treatment with EcoHIV, a modified strain of HIV-1 NL4-3 generated by replacing the coding regions of gp120 with the envelope of ecotropic murine leukemia virus (gp80; Potash et al., 2005; Saini et al., 2007). While EcoHIV does not infect human cells, the virus was propagated in HEK293T/17 cells using the same methodology as HIV. Throughout the study, we did not observe any impact of EcoHIV on human cells, indicating that the responses in HIV-infected pericytes were specific and not influenced by general cellular stressors (i.e., pro-inflammatory cytokines or chemokines) which are typically present in viral stocks employed for pericyte infection.

HIV infection of BBB pericytes demonstrated a dual-stage response pattern, the first stage being an increase in viral replication occurring at days 2–3 post-infection, followed by the second stage of a steady decrease in active viral production that reaches a minimum at days 7–8 post-infection (Figure 1A). Our recently published study (Castro et al., 2016) linked this pattern of changes to the alterations of intracellular occludin levels. Interestingly, integration of HIV DNA continued to increase while the first active phase of viral production ceased (Figure 1B), suggesting that human BBB pericytes may play a role in the formation of viral reservoirs in HIV-infected brains. Thus, BBB pericytes, in addition to perivascular macrophages, microglia or astrocytes (Cosenza et al., 2002; Churchill et al., 2009; Schnell et al., 2011; Gray et al., 2014; Joseph et al., 2015; Marban et al., 2016), may be another cell type in the brain that harbors dormant HIV infection. Such a role is important because HIV reservoirs in the CNS have a bystander impact on cellular dysfunction and may be responsible for preserving the production of neurotoxic factors and chronic inflammatory status in the CNS (Gorry et al., 2003; Eugenin and Berman, 2007; Eugenin et al., 2011; Narasipura et al., 2014). Nevertheless, more detailed work is required to properly address the formation of HIV reservoirs in BBB pericytes as well as their possible reactivation in response to molecular or cellular stressors.

Novel results of the current study also demonstrate that a prominent GJ protein, Cx43, participates in HIV infection of BBB pericytes. Protein levels of total Cx43 were significantly up-regulated and correlated with the time of active HIV infection. In addition, expression of pCx43, an important player in regulating GJ trafficking, assembly/disassembly, degradation and gating (Lampe and Lau, 2004; Solan and Lampe, 2009; Axelsen et al., 2013), was proportional to the up-regulated total Cx43. Cx43 expression was also elevated in BBB pericytes in EcoHIV-infected mice. While these *in vivo* results are supportive, a careful investigation on involvement of GJs and HIV entry via gp120 and co-receptors-mediated signaling pathway is required to better understand impact of HIV infection on GJs due to the lack of gp120/gp41 engagement in EcoHIV infection. Furthermore, GJ-mediated intercellular communication was elevated in HIV-infected BBB pericytes, and correlated with the peak of infection. These results suggest highly unique responses in HIV-infected BBB pericytes because Cx43 was not altered and GJ function was decreased in HIV-infected human astrocytes (Eugenin and Berman, 2007).

We then investigated the role of occludin in HIV-induced alterations of Cx43 and GJ-mediated intercellular communication. The rationale of these experiments was related to our findings that occludin levels regulate HIV transcription rates in pericytes, namely, a decrease in occludin stimulates robust HIV replication (Castro et al., 2016). This effect is long standing as demonstrated in Figure 5A. Silencing occludin resulted in marked down-regulation of Cx43 and functional GJ-mediated dye-coupling (Figures 5C,E, respectively). Such effects are caused by the direct binding of occludin to another tight junction protein, ZO-1, which is known to interact with Cx43. In fact, specific domains of Cx43, such as the ser(-9) and ser(-10) at the C-terminal, serve as binding sites for ZO-1 (Schmidt et al., 2001; Xiao et al., 2011). On the other hand, the impact of occludin silencing on Cx43 protein levels and reduced GJ channel function was completely reversed by HIV infection. Indeed, the levels of Cx43 and GJ-mediated dye coupling were equal in the HIV-infected and HIV-infected plus occludin silencing groups, and markedly elevated as compared to NI controls (Figures 5D,E, respectively). These results confirm a strong regulatory impact of HIV infection on GJ function in BBB pericytes. Recent studies suggest that reverse transcription of HIV is affected by the levels of a nucleocytoplasmic shuttling protein, human antigen R (HuR; or ELAVL1), which also regulates GJs and GJ-mediated intercellular communication (Lemay et al., 2008; Ale-Agha et al., 2009). The involvement of HuR in the early steps of HIV replication might influence the levels of Cx43 and GJ-mediated dye coupling as observed in the present study.

Previous studies reported that GJs of HIV-infected astrocytes spread to neighboring uninfected cells via cytochrome c-mediated inositol trisphosphate and calcium ions, resulting in disruption of BBB integrity with extensive neuroinflammation (Eugenin and Berman, 2007, 2013; Eugenin et al., 2011). On the other hand, the current study demonstrates that GJs play an important role in the propagation of HIV infection in BBB pericytes. This is supported by the observation that the GJ blocker, CBX, drastically abolished not only dye-coupling in neighboring cells (Figure 4D), but also p24 production in infected cultures (Figure 6). This effect was observed both in WT and occludin-depleted pericyte cultures. Thus, these observations strongly support the notion that GJ-mediated intercellular communication is involved in propagation of HIV infection to neighboring cells.

In addition to GJs, cells communicate via single-membrane channels, also known as HCs, to allow small signaling molecules to be released into the extracellular space. The molecules involved in this paracrine communication include glutamate, aspartate, ATP, and prostaglandins (Orellana et al., 2009). HIV-infected human pericytes, both WT and occludin-depleted, exhibited a substantial increase in extracellular ATP levels, suggesting stimulation of functional hemichannel activity. In addition, this effect was abrogated in the presence of CBX (Figure 7), indicating the involvement of Cx-containing HCs. ATP release into the extracellular milieu may be an additional factor affecting HIV infection via possible activation of purinergic receptors (Velasquez and Eugenin, 2014; Graziano et al., 2015; Swartz et al., 2015). These receptors mediate nucleosides and nucleotides, including adenosine and ATP, and play a crucial role in the innate and adaptive immune responses (Paoletti et al., 2012; Cekic and Linden, 2016). Indeed, it has been shown that HIV-induced extracellular ATP release and purinergic receptors actively participate in HIV uptake by human macrophages (Hazleton et al., 2012; Tewari et al., 2015).

## Conclusion

Our results indicate the role of Cx43 and intercellular communication mediated by GJs and Cx-containing HCs in HIV infection of human BBB pericytes. These results contribute to a better understanding of the intercellular network activated by HIV, which may not only affect the BBB, but also contribute to the CNS dysfunction and neurocognitive changes frequently observed in HIV-infected brains. In addition, the presented findings may provide a foundation for novel treatments to ameliorate and protect the BBB from dysfunction in HIV CNS infection.

## Author Contributions

HJC and MT designed the study and wrote the manuscript. HJC, AMK and LB performed all the experiments, collected the data and analyzed the data. All authors reviewed the manuscript.

## Conflict of Interest Statement

The authors declare that the research was conducted in the absence of any commercial or financial relationships that could be construed as a potential conflict of interest.
