# Supplementary material for: HIV Alters Gap Junction-Mediated Intercellular Communication in Human Brain Pericytes
Source: Front Mol Neurosci. 2017 Dec 12;10:410. doi: 10.3389/fnmol.2017.00410 (PMC5732912; doi:10.3389/fnmol.2017.00410)
Supplement: Supplementary file 1 [file Presentation_1.pdf]

*Supplementary Material*

# HIV alters gap junction-mediated intercellular communication in human brain pericytes

**Hyung Joon Cho, Alyce Mei-Shiuan Kuo, Luc Bertrand, and Michal Toborek***

*** Correspondence:**
Dr. Michal Toborek,
Department of Biochemistry and Molecular Biology,
University of Miami School of Medicine
Gautier Bldg., Room 528
1011 NW 15th Street
Miami, FL 33136
Phone# 305-243-0230
mtoborek@med.miami.edu

# 1 Supplementary Data

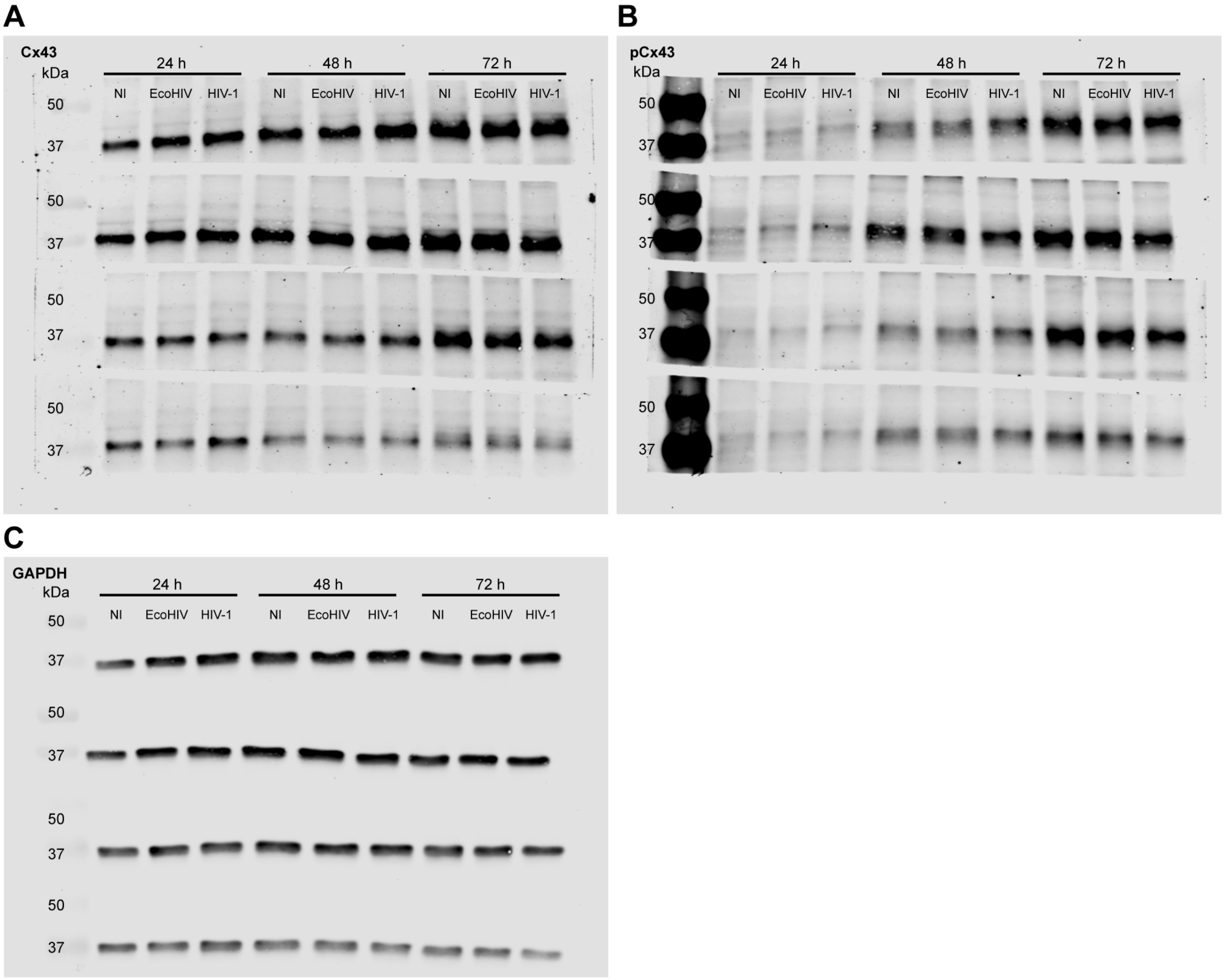


**Supplementary Figure S1**. A representative set (n=4) of full size blot used in Figure 2A to detect Cx43 (A), pCx43 (B), and GAPDH (C). A single blot was cut at molecular weight marker from 30 to 60 kDa to maximize immunoreactivity of primary antibodies for target proteins. NI: non-infected; Cx43: connexin 43; pCx43: phosphorylated Cx43.

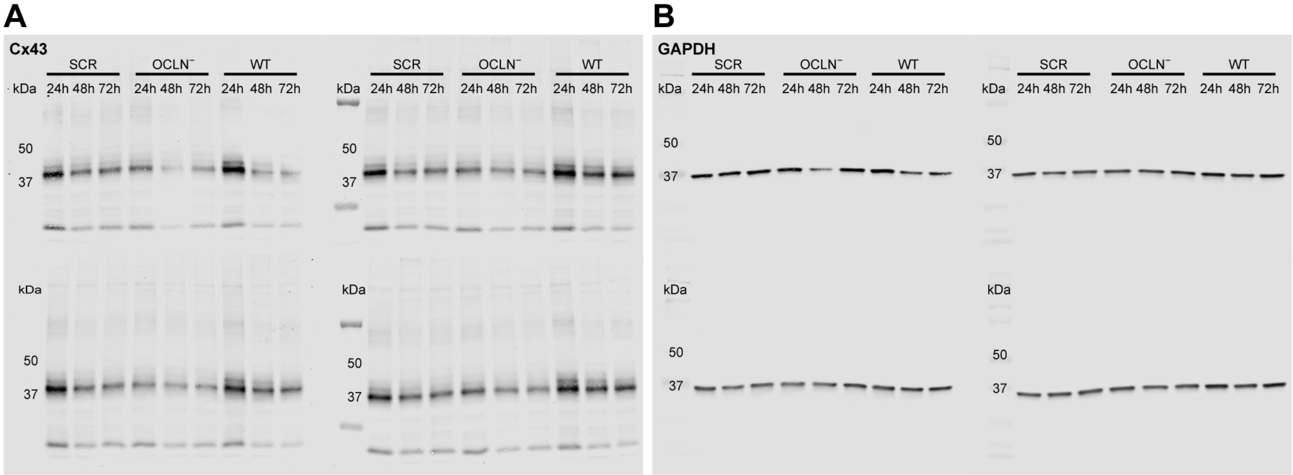


**Supplementary Figure S2**. A representative set (n=4) of full size blot used in Figure 5C to detect Cx43 (A) and GAPDH (B). SCR: scrambled; $OCLN^{-}$: occludin depleted; WT: wild type.

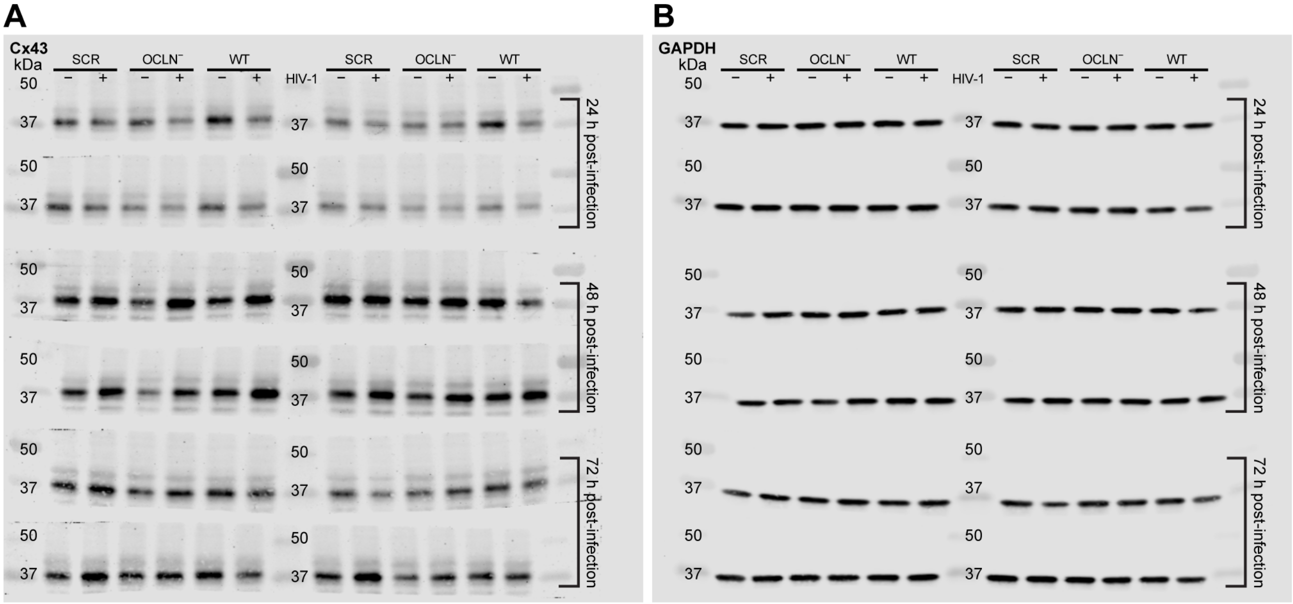


**Supplementary Figure S3**. A representative set (n=4) of full size blot used in Figure 5D to detect Cx43 (A) and GAPDH (B). A single blot was cut at molecular weight marker from 30 to 60 kDa to maximize immunoreactivity of primary antibodies for target proteins.
